# *Sesbania Mosaic Virus* (SeMV) Infectious Clone: Possible Mechanism of 3′ and 5′ End Repair and Role of Polyprotein Processing in Viral Replication

**DOI:** 10.1371/journal.pone.0031190

**Published:** 2012-02-15

**Authors:** Kunduri Govind, Kristiina Mäkinen, Handanahal S. Savithri

**Affiliations:** 1 Indian Institute of Science, Bangalore, Karnataka, India; 2 University of Helsinki, Helsinki, Finland; Kantonal Hospital St. Gallen, Switzerland

## Abstract

*Sesbania mosaic virus* (SeMV) is a positive stranded RNA virus belonging to the genus *Sobemovirus*. Construction of an infectious clone is an essential step for deciphering the virus gene functions *in vivo*. Using *Agrobacterium* based transient expression system we show that SeMV icDNA is infectious on *Sesbania grandiflora* and *Cyamopsis tetragonoloba* plants. The efficiency of icDNA infection was found to be significantly high on *Cyamopsis* plants when compared to that on *Sesbania grandiflora*. The coat protein could be detected within 6 days post infiltration in the infiltrated leaves. Different species of viral RNA (double stranded and single stranded genomic and subgenomic RNA) could be detected upon northern analysis, suggesting that complete replication had taken place. Based on the analysis of the sequences at the genomic termini of progeny RNA from SeMV icDNA infiltrated leaves and those of its 3′ and 5′ terminal deletion mutants, we propose a possible mechanism for 3′ and 5′ end repair *in vivo*. Mutation of the cleavage sites in the polyproteins encoded by ORF 2 resulted in complete loss of infection by the icDNA, suggesting the importance of correct polyprotein processing at all the four cleavage sites for viral replication. Complementation analysis suggested that ORF 2 gene products can act in *trans*. However, the *trans* acting ability of ORF 2 gene products was abolished upon deletion of the N-terminal hydrophobic domain of polyprotein 2a and 2ab, suggesting that these products necessarily function at the replication site, where they are anchored to membranes.

## Introduction

**Figure 1:**
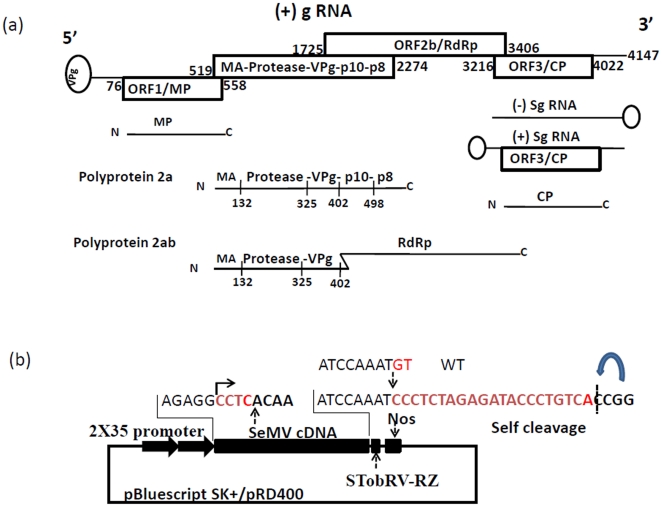
Genome organization. (a) SeMV is a single stranded RNA virus with genome size of 4147 nt. The 5′ end of the genome is covalently linked to VPg and the 3′ end lacks polyA tail. ORF 1 encodes movement protein and ORF 3 encodes the coat protein which is expressed through a subgenomic RNA (sgRNA). The ORF 2 codes for two polyproteins 2a and 2ab. The numbers indicate the position of start and stop codons in each of the ORFs. The polyprotein 2a contains N-terminal membrane anchor (MA)-protease-VPg-p10-p8 domains. The polyprotein 2ab contains N-terminal membrane anchor (MA)-protease-VPg-RdRp. The RdRp is expressed through a −1 ribosomal frame shifting mechanism. The numbers in the polyproteins 2a and 2ab indicate the cleavage site positions. (b) Features of infectious clone: The infectious construct was initially made in pBluescript SK+ vector and later subcloned into pRD400 vector. The infectious construct consists 2×35S Promoter-SeMV cDNA-sTobRV RZ (Ribozyme)-Nos terminator. It has additional 4 nt at the 5′ end (5′ CCTC 3′) and 21 nt at the 3′end. The 3′ terminal two nucleotides present in the wild type viral RNA are absent in this clone (5′ AAA T 3′ instead of 5′ AAA TGT 3′). The ribozyme self cleavage site is shown by a curved arrow.

*Sesbania mosaic virus* (SeMV) is a member of the genus *Sobemovirus*. The viruses from this genus infect both mono and dicotyledonous plants [1], [2]. However, their host range is narrow and each virus infects only a small number of monocots or dicots. The natural host for SeMV is *Sesbania grandiflora*; however it can also infect *Cyamopsis tetragonoloba* (guar bean or cluster bean), which is an experimental host [3]. Both these host plants are dicotyledonous and belong to *Leguminosae* family. SeMV is a single stranded positive sense RNA virus with a genome size of 4147 nt (Fig. 1a). The 5′ end of the genome is covalently linked to a viral protein genome linked (VPg) and the 3′ end lacks the poly A tail [2]. *Sobemoviruses* encode 3 open reading frames (Fig. 1a). The 5′ proximal ORF (ORF 1) codes for the movement protein (MP) which is involved in cell to cell movement of the virus and is a suppressor of post transcriptional gene silencing [4], [5], [6], [7], [8], [9]. The 3′ proximal ORF (ORF 3) is translated into coat protein (CP) from a subgenomic RNA (sgRNA) generated during replication (Fig. 1a). CP is a major structural protein that forms T = 3 icosahederal capsids. In addition, the CP is shown to be important for virus movement [1], [5], [10]. ORF 2 is translated by a leaky scanning mechanism and codes for two polyproteins 2a and 2ab. It was demonstrated that SeMV polyprotein 2a has a domain arrangement of membrane anchor (MA)-protease-VPg-p10-p8 [11]. The polyprotein 2ab that is translated by a −1 ribosomal frame shift mechanism has a domain arrangement of MA-protease-VPg-RdRp [11], [12]. The polyproteins 2a/2ab were predicted to contain an N-terminal transmembrane domain (70 residues from N-terminus) and a cleavage site was identified at residue 132 [11], [13]. Both VPg and p8 are intrinsically disordered domains that influence the activity of the neighbouring folded domains, namely protease and p10 respectively [14], [15]. For example, it was shown that the protease-VPg (Δ70 Pro-VPg) but not the protease (Δ70 Pro) alone is active [15]. Similarly, the ATPase activity of p10 domain was stimulated by the p8 domain present at its C-terminus [14]. Further, VPg-RdRp is the predominant intermediate of 2ab processing in *E.coli* [11]. However, it was demonstrated that the recombinant RdRp domain by itself possesses RNA structure dependent and primer independent RNA polymerase activity [16]. Majority of these studies were performed using *in vitro*/*ex vivo* methods and it is therefore essential to establish these functions *in vivo* for better understanding of the biology of *Sobemoviruses*.

Inoculation of *in vitro* transcripts from full length cDNA clones onto whole plants or protoplasts is the common strategy used for studying *in vivo* functions of the viral encoded proteins [17], [18]. However use of DNA based *Agrobacterium*-mediated transient expression in planta is a better alternative as the transcripts synthesized *in vivo* are much more stable [19]. The agroinfiltration is simple, efficient, and widely used [20], [21], [22], [23]. It involves delivery of genes of interest from *Agrobacterium* containing a Ti- plasmid into the plant cell nucleus followed by transcription of the genes [19], [24], [25]. The full length transcripts thus generated could then enter the cytosol and express the viral encoded proteins enabling the replication of the viral RNA and subsequent steps of the viral life cycle [19].

This paper describes the construction of SeMV full length infectious cDNA (icDNA) clone in a binary vector and optimization of conditions for *Agrobacterium* mediated transient expression of SeMV RNA leading to infection. Based on the observed 5′ and 3′ end sequences of SeMV progeny genomic RNAs (gRNA) from different SeMV icDNA mutants and presence of various forms of VPg, a possible mechanism for genome end repair *in vivo* is proposed. Mutational analysis of cleavage sites in the polyproteins encoded by ORF2 showed that all the four cleavage sites identified earlier [11] are crucial for SeMV infection *in vivo*. Further, coinfiltration analysis showed that proteins encoded by ORF 2 but not NΔ70 ORF2 could act *in trans* and support the replication of cleavage site mutants.

## Results

### Features of SeMV full length cDNA clone

Initially the full length cDNA construct of SeMV was generated in pBluescript SK+ vector through a series of cloning steps, resulting in a clone with 2×35S promoter-SeMV full length cDNA-sTobRV Ribozyme-Nos terminator (Fig. 1b & Fig.S1). This cassette was then cloned into pRD400 binary vector and the clone was named SeMV icDNA. The cloning strategy resulted in the addition of 4 nucleotides at the 5′ end and 21 nucleotides at the 3′ end of the SeMV cDNA. Further, this clone had two nucleotides less at the 3′ end when compared to wild type genome (5′ AAAT 3′ instead of 5′ AAATGT 3′) (Fig. 1b).

### Agroinfiltration on *Sesbania grandiflora*

**Figure 2:**
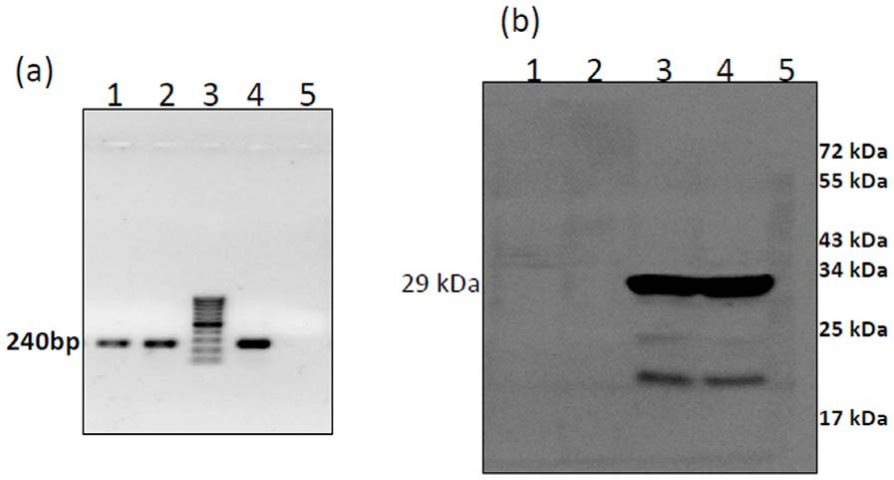
RT-PCR and Western blotting analysis of *Sesbania grandiflora* infiltrated with SeMV icDNA. (a) RT-PCR of total RNA isolated from systemically infected *Sesbania* leaves 21 dpi with SeMV icDNA: The RT-PCR was carried out with SeMV RdRp reverse and coat protein forward primer as described in the methods section. lanes 1 & 2, two different plants infiltrated with SeMV icDNA, lane 3, 100 bp ladder, lane 4, RT-PCR with SeMV genomic RNA, lane 5, RT(−)-control. (b) Western blot analysis of *Sesbania* plants *Agrobacterium* infiltrated with SeMV icDNA clone using CP specific antibodies: lanes 1 and 2 correspond to mock agroinfiltrated *Sesbania* leaf samples; lanes 3 and 4 leaf extracts of systemically infected leaves 21 dpi; lane 5 protein molecular mass marker.

In order to check the transient expression and infectivity of SeMV icDNA, agroinfiltration was carried out on *Sesbania grandiflora* plants (the natural host) as described in the methods section. Interestingly, 2–3 weeks post infiltration, about 5% of the plants showed symptoms similar to that of wild type SeMV infection. RT-PCR analysis of total RNA extracted from systemic leaves showed the presence of the expected 240 bp product (Fig. 2a lanes 1 & 2) with the same mobility as that obtained with viral RNA template (Fig. 2a lane 4). Further, western blot analysis with CP specific antibodies confirmed the presence of SeMV CP in systemically infected leaves (Fig. 2b lanes 3 & 4). Plants which did not show symptoms were negative to RT-PCR and western blot analysis with CP specific antibodies (Fig. 2a lane 5 & 2b lanes 1 & 2 respectively). Leaf extracts of SeMV icDNA infected plants induced mosaic symptoms on fresh plants showing that progeny virus from SeMV icDNA infected plants behaved like the wild type virus. These results suggest that SeMV icDNA could mimic the wild type virus.

### Agroinfiltration on *Cyamopsis tetragonoloba* plants

**Figure 3:**
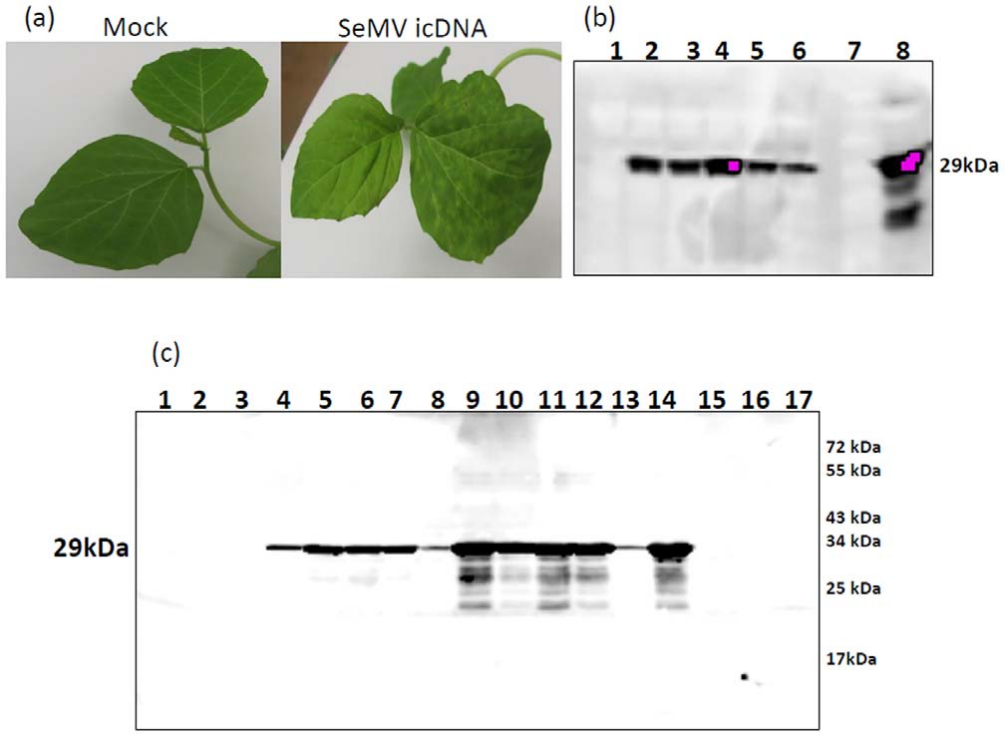
Agroinfiltration of SeMV icDNA on *Cyamopsis tetragonoloba* plants and analysis of time course of SeMV icDNA infection. (a) Mock and SeMV icDNA infiltrated *Cyamopsis* leaves (b) Western blot of SeMV icDNA infected *Cyamopsis tetragonoloba* plants. Lane 1, mock agroinfiltrated leaf extract; lanes 2–6, SeMV icDNA infected systemic leaves from five independent plants showing symptoms; lane 7, protein molecular mass markers; lane 8, positive control (native virus infected leaf extract). (c) Time course of SeMV icDNA infection on *Cyamopsis* plants: western blot analysis using CP antibodies. Lanes 1–3, 3 dpi; lanes 4–8, 6 dpi; lanes 9–13, 9 dpi; lane 14, is a positive control; lanes 15–16 are mock agroinfiltrated samples; lane 17 is a protein molecular mass marker.

As the efficiency of SeMV icDNA infection was rather low on *Sesbania* plants, the SeMV icDNA was tested for its ability to infect another experimental host, namely *Cyamopsis tetragonoloba* [3]. Initially agroinfiltration was carried out with transformed agrobacteria of cell density 0.6 at 600 nm (OD_600_). Symptom appearance was monitored 2–3 weeks post infiltration. Interestingly, about 50% of the agroinfiltrated *Cyamosis* plants developed local chlorotic spots and necrotic lesions on the systemically infected leaves (Fig. 3a). These symptoms were identical to that of the wild type SeMV infection on these plants. Western blot analysis confirmed the presence of CP in the systemic leaves showing symptoms (Fig. 3b lanes 2–6). When total leaf extracts from these plants was used to inoculate fresh plants, symptoms similar to that obtained with wild type virus were observed. Since *Cyamopsis* gave better efficiency of infection with SeMV icDNA, all further experiments were carried out on these plants.

In order to optimize the efficiency of SeMV icDNA infection, agroinfiltration was carried out with different densities of the cells (OD_600_ 0.2, 0.4, 0.6, 0.8, 1). It was observed that with increase in cell density, there was an increase in the number of plants infected (data not shown). About 80% of the plants showed infection when infiltrated with cells of density OD_600_ 0.8. However, further increase in cell density to 1.0 or more did not result in 100% infection.

Agroinfiltration analysis with pEAQ-GFP showed that the GFP expression was low in *Sesbania* plants when compared to *Cyamopsis* plants (Fig.S2). This could be due to inefficient T-DNA transfer by *Agrobacterium* in *Sesbania* plants. Therefore lack of efficient T-DNA transfer in *Sesbania* plants could be one of the reasons for observed difference in infectivity of the two plants. Lack of 100% infectivity in *Cyamopsis* could be due to difference in resistance from plant to plant.

To check the time course of virus accumulation, *Cyamopsis* cotyledons were infiltrated with *Agrobacterium* carrying SeMV icDNA at OD_600_ 0.8. The infiltrated cotyledons were collected from different plants at 3, 6, and 9 dpi and subjected to western blot analysis as described in the methods section. As shown in Fig. 3c, CP was not detected at 3 dpi (lanes 1–3, represent plant numbers) at which time probably the replication of the viral genome and synthesis of subgenomic RNA was initiated. However, CP could be detected in good amount at 6 dpi (Fig. 3c lanes 4–8) which increased further at 9 dpi (lanes 9–13). Among the plant samples analyzed, two (lane 8 and 13) showed only a faint band for CP. Mock infiltrated plants (lanes 15 and 16) did not show the presence of CP at 9 dpi and the CP from native virus (Fig. 3c lane 14) migrated at the same position as the CP in the samples from icDNA infiltrated plants (positive control). The minor bands below the intact CP (Fig. 3c lane 9–14) could be due to degradation of CP during extraction. The time course analysis of plants mechanically inoculated with native virus also gave similar pattern of CP expression (data not shown).

**Figure 4:**
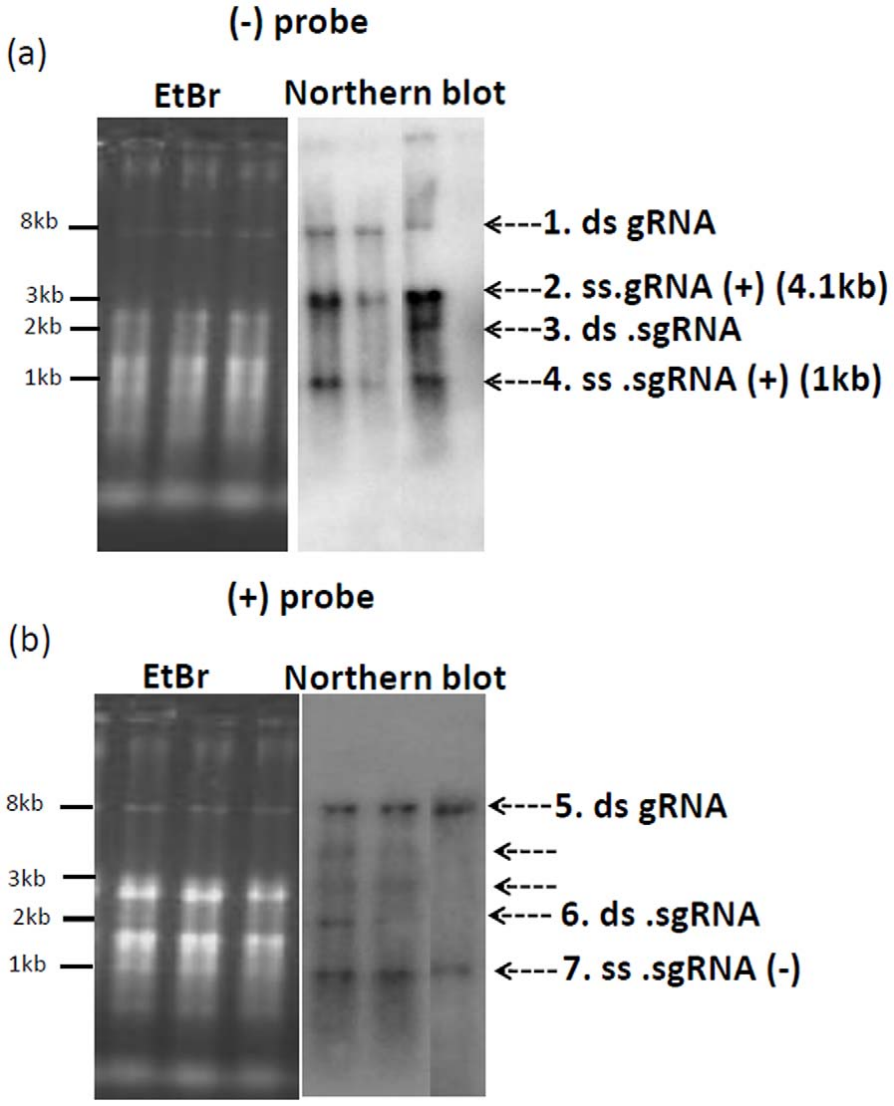
Northern analysis of SeMV icDNA infiltrated cotyledon leaves of *Cyamopsis tetragonoloba* plants. (a) 0.8% TBE agarose gel (EtBr staining) and northern blot analysis of total RNA extracted from SeMV icDNA agroinfiltrated leaves 6 dpi from three different plants. The negative sense ^32^P labelled probe used for hybridization was complementary in sequence to the (+) sgRNA. (b) 0.8% TBE agarose gel analysis (EtBr staining) and northern blot analysis of total RNA extracted from SeMV icDNA agroinfiltrated leaves 6 dpi. The positive sense ^32^P labelled probe corresponding in sequence to that of (+) sgRNA was used for hybridization.

Northern analysis was carried out to detect the viral RNA species present in SeMV icDNA infiltrated cotyledon leaves. Viral RNA could not be detected with either the negative or positive sense probes at 3 dpi (data not shown). However, at 6 dpi samples showed double stranded (ds) gRNA, single stranded (ss) (+) gRNA, ds sgRNA and ss (+) sgRNA when hybridized with (−) sgRNA probe as estimated from the sizes of the positive signals (Fig. 4a). The ds gRNA was also detectable in EtBr stained gel (Fig. 4 a & b). Similarly when the hybridization was carried out with (+) sgRNA probe, ds gRNA, ds sgRNA and ss (−) sgRNA could be detected. However, ss (−) gRNA was not detectable (Fig. 4b). Lack of signal for ss (−) gRNA could be due to its low abundance or its presence predominantly in the ds replicative form. In two of the samples, a faint band corresponding to 2.7 and 6 kb was observed in the blot (Fig. 4b). The identities of these bands are unclear.

**Figure 5:**
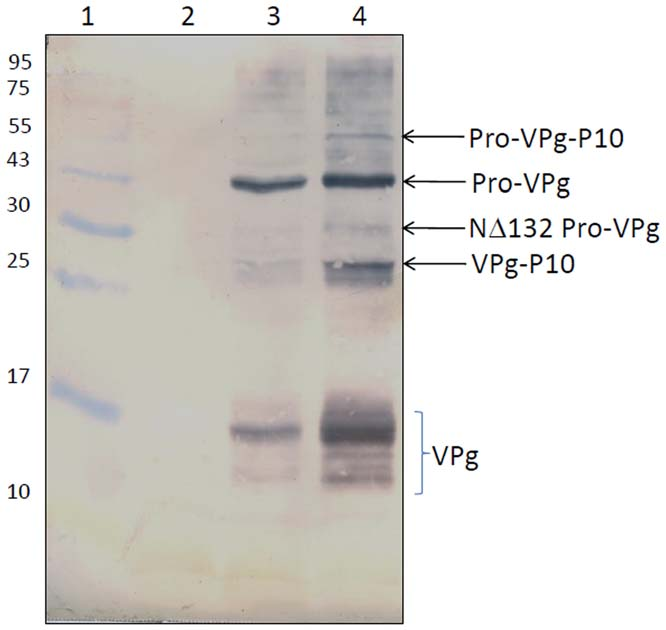
Western blot analysis with membrane enriched fraction of SeMV icDNA infected cotyledon leaves. Western blot analysis with SeMV VPg polyclonal antibodies: Leaf samples were collected at 9 dpi; Lane 1, protein molecular weight marker; Lane 2, crude membrane fraction (25,000 g) from mock infiltrated cotyledon leaves. Lanes 3 & 4, Crude membrane fractions obtained at 10,000 g and 25,000 g respectively from SeMV icDNA infiltrated cotyledon leaves.

Western blot analysis was carried out with VPg antibodies to detect the nonstructural proteins in the SeMV icDNA infiltrated cotyledon leaf extracts. The crude membrane fraction was used for this analysis to enrich the viral proteins. As shown in Fig. 5. lane 3 (10,000 *g* membrane fraction) & lane 4 (25,000 *g* membrane fraction) specific bands corresponding in size to 54 kDa, 43 kDa, 29 kDa and 27 kDa were observed, which could correspond to Pro-VPg-p10, Pro-VPg, NΔ132 Pro-VPg and VPg-p10 respectively. In addition, bands with molecular weight ranging from 12–17 kDa were also observed with a prominent band at 16 kDa. It may be noted that the expected molecular mass of the VPg is 9 kDa and the *E.coli* expressed VPg does not move abnormally on SDS-PAGE [15], [26]. This observed abnormal mobility of VPg could be due to post-translational modifications of VPg *in planta*. Such an abnormal migration of VPg, due to post-translational modifications, has also been reported in other members [27], [28] of the genus *Sobemovirus*. Mass spectrometric analysis of CfMV, RYMV, SBMV and RGMoV VPgs isolated from native virus showed that they were phosphorylated and nucleotidylylated [28], [29]. The Pro-VPg and VPg bands could also be detected in native virus inoculated cotyledon leaf membrane fraction (data not shown). Over all, the western blot analysis revealed that various processed forms of polyprotein 2a and post-translationally modified forms of the VPg are detectable in membrane enriched fractions.

### The 3′ and 5′ end repair of SeMV genome *in vivo*

**Figure 6:**
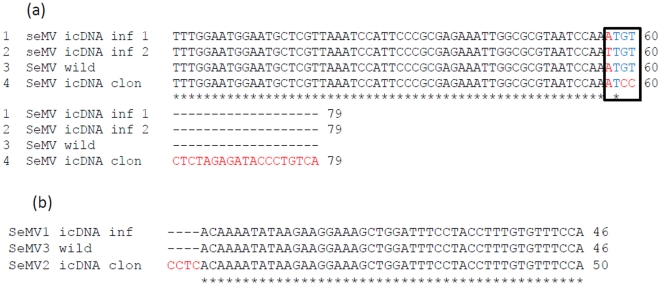
*In vivo* 3′ end repair of SeMV icDNA. (a) Comparison of nucleotide sequence corresponding to the 3′ end of the genomic RNA from native virus (SeMV Wild type), genomic RNA from virus purified from SeMV icDNA infiltrated sample (SeMV icDNA inf 1 & 2 ) and sequence of SeMV icDNA clone (SeMV icDNA clone). (b) Comparison of nucleotide sequence corresponding to the 5′ end of the genomic RNA from native virus (SeMV wild type), genomic RNA from virus purified from SeMV icDNA infiltrated sample (SeMV icDNA inf) and sequence of SeMV icDNA clone (SeMV icDNA clone).

Due to the cloning strategy used, SeMV icDNA had 4 additional nucleotides at the 5′ end and 21 nucleotides at the 3′ end and there was a 2 nt deletion at the 3′ end when compared to the wild type SeMV sequence. It was therefore of interest to determine the 3′ and 5′ sequence of the viral RNA obtained after icDNA infection. The virions were purified from SeMV icDNA infected plants and the viral RNA was extracted. The RNA was poly A tailed and reverse transcribed with oligo dT primer and subsequently amplified using 3′UTR forward (Table 1) and oligo dT reverse primers. The PCR product was cloned and sequenced to identify the 3′ terminal nucleotide sequence of SeMV. Similarly, to determine the 5′ terminal sequence of SeMV, cDNA was synthesized using progeny viral RNA as template and P1 reverse primer (Table 1), poly dA was added at the 3′ end of cDNA and the second strand synthesis and amplification was carried out with oligo dT and P1 reverse primers (Table 1) as described in the methods section. The PCR product was cloned at *SmaI* site of pBluescript SK+ vector and sequenced. The sequencing result showed that the 5′ and 3′ ends of the progeny viral RNA did not contain the extra nucleotides (Fig. 6 a & b). Further the 3′ end sequence was found to be either 5′ A TGT 3′ or 5′ T TGT 3′ (Fig. 6 a). It may be noted that the later sequence differs from the wild type sequence at the 4^th^ nucleotide from the 3′ end (Fig. 6a). Together these observations suggest that during replication of the transcripts generated from icDNA, the extra nucleotides were removed and the 3′ end sequence was repaired. Further, the virus was isolated from infiltrated leaves and systemic leaves independently, RNA extracted and RT-PCR was carried out with 3′ UTR antisense and 5′ UTR sense primers. The PCR product was cloned into pBluescript SK+ vector and sequenced. The sequencing results from two independent clones (one from infiltrated leaves and second from systemic leaves) showed that except for the differences at the extremity of the genome there were no other changes in the sequence (data not shown).

**Table 1:** Description of oligonucleotides used in the study.

Name	Sequence (5′-3′)	Description
35S FW	5′ CGGGATCCGATCTCCTTTGCCCCGGAGATC 3′	To PCR amplify 35S promoter; the BamHI site is under lined.
35S Rev	5′ CCTCTCCAAATGAAATGAACTTCCTTATATAG 3′	
sTobRV RZ FW	5′ TCTAGAGATACCCTGTCACCGGAT 3′	To PCR amplify the sTobRV ribozyme; the XbaI and StuI sites are underlined in FW and Rev primers respectively.
sTobRV RZ Rev	5′AGGCCTCTGCAGGACAGTC 3′	
5′UTR FW	5′ CTAAGGCCTCACAAAATATAAGAAGGAAAGCTG 3′	To PCR amplify the SeMV full length cDNA; the StuI and SmaI sites are underlined.
3′UTR Rev	5′ CCCCCGGGATTTGGATTACGCGCCAATTTCTC 3′	
Nos FW	5′ TATACCGGTCCCGATCTAGTAACATAGATG 3′	To PCR amplify the Nos terminator; the AgeI and KpnI sites are underlined in FW and Rev primers respectively.
Nos Rev	5′ ATATGGTACCGATCGTTCAAACATTTGG 3′	
E132A FW	5′ GATGCTTCCAATGCGTCAGCTGTGTTGGGG 3′	To introduce E132A mutation in SeMV icDNA; the restriction site PvuII created is underlined.
E132A Rev	5′ CCCCAACACAGCTGACGCATTGGAAGCATC 3′	
E325A FW	5′ CTCTTAAGATCTAATGCGACTCTCCC 3′	To introduce E325A mutation in SeMV icDNA, the restriction site BglII created is underlined.
E325A Rev	5′ GGGAGAGTCGCATTAGATCTTAAGAG 3′	
E402A FW	5′ GAAAACGCTCAAGCTTCCGTCGCTGTTGAG 3′	To introduce E402A mutation in SeMV icDNA; the restriction site HindIII created is underlined.
E402A Rev	5′ CTCAACAGCGACGGAAGCTTGAGCGTTTTC 3′	
E498A FW	5′ GTTATTACAAGCAGGCAAGTTTAATCCTTCCAG 3′	To introduce E498A mutation in SeMV icDNA.
E498A Rev	5′ CTGGAAGGATTAAACTTGCCTGCTTGTAATAAC 3′	Mutation was confirmed by sequencing.
5′U TR Δ 1 nt FW	5′ CATTTCATTTGGAGAGGCCCCAAAATATAAGAAGGAAAG 3′	Used for deletion of one nucleotide from the 5′ end, StuI site abolished is underlined.
5′UTR Δ 1 nt Rev	5′ CTTTCCTTCTTATATTTTGGGGCCTCTCCAAATGAAATG 3′	
5′UTR Δ 3 nt FW	5′ CATTTCATTTGGAGAGGCCCCAAATATAAGAAGGAAAG 3′	Used for deletion of 3 nucleotides from the 5′ end StuI site abolished is underlined.
5′UTR Δ 3 nt Rev	5′ CTTTCCTTCTTATATTTGGGGCCTCTCCAAATGAAATG 3′	
5′UTR Δ 5 nt FW	5′ CATTTCATTTGGAGAGGCCCCATATAAGAAGGAAAG 3′	Used for deletion of 5 nt from the 5′ end, StuI site abolished is underlined.
5′UTR Δ 5 nt Rev	5′ CTTTCCTTCTTATATGGGGCCTCTCCAAATGAAATG 3′	
3′UTR Δ 3 nt FW	5′ GGCGCGTAATCCAAACCCACTAGAGATACCCTGTC 3′	Used for deletion of 3 nt from the 3′ end, XbaI site abolished is underlined.
3′UTR Δ 3 nt Rev	5′ GACAGGGTATCTCTAGTGGGTTTGGATTACGCGCC 3′	
3′UTR Δ 4 nt FW	5′ GGCGCGTAATCCAACCCACTAGAGATACCCTGTC 3′	Used for deletion of 4 nt from the 3′ end, XbaI site abolished is underlined.
3′UTR Δ 4 nt Rev	5′ GACAGGGTATCTCTAGTGGGTTGGATTACGCGCC 3′	
3′UTR Δ 5 nt FW	5′ GGCGCGTAATCCACCCACTAGAGATACCCTGTC 3′	Used for deletion of 5 nt from the 3′ end, XbaI site abolished is underlined.
3′UTR Δ 5 nt Rev	5′ GACAGGGTATCTCTAGTGGGTGGATTACGCGCC 3′	
ORF2 FW	5′ CTAGCTAGCCATATGTATCATCCGAGCTGCAAGG 3′	To PCR amplify the SeMV ORF 2.
NΔ70 ORF2 FW	5′ CCCCATATGGAGGCAAAGCAGGACAG 3′	To PCR amplify the SeMV NΔ70 ORF 2.
RdRp/ORF 2 Rev	5′ CGGGATCCTTACGAATCCGCACCATAGC 3′	Used in RT-PCR and to amplify ORF 2.
3′UTR FW	5′ AAC CAA CTG CCT CAG CCC TG 3′	To PCR amplify the polyA tailed 3′ UTR cDNA.
CP sense	5′ GGG GAA TAC TCC ATC GCC CC 3′	Used in RT-PCR reaction.
P1 Rev	5′ CCGCCGAAGCTTGAATTCCGGCCCGTTTTCACAAGGAGC 3′	Used for 5′ RACE analysis.
Oliogo dT	5′ TTT TTT TTT TTT TTT TTT 3′	To PCR amplify the polyA tailed 3′ UTR cDNA.
T7 FW	5′ TAATACGACTCACTATAGGG 3′	Used in cloning and sequencing of SeMV icDNA.
T3 FW	5′AATTAACCCTCACTAAAGGGA 3′	

### Mutational analysis of 5′ and 3′ end nucleotides of SeMV genome

**Figure 7:**
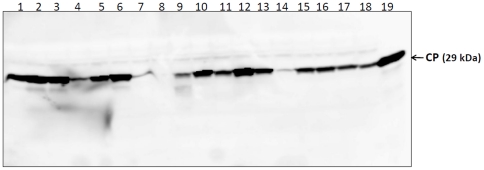
Mutational analysis of 5′ and 3′ terminus of SeMV genome. (a) Western blot analysis of 3′ and 5′ end deletion mutants of SeMV icDNA infiltrated plants. Lanes 1–3 represent 3′ UTR Δ3 nt icDNA; lanes 4–6 corresponds to 3′UTR Δ4 nt icDNA; lanes 7–9 represent 3′UTR Δ5 nt icDNA; lanes10–12 corresponds to 5′UTR Δ1 nt icDNA; lanes 13–15 represents for 5′UTR Δ3 nt icDNA; lanes 16–18 corresponds to 5′UTR Δ5 nt icDNA and lane 19 is a positive control (SeMV native virus).

The 3′ end of the progeny viral RNA sequence obtained after repair 5′ TGT 3′/5′ T TGT 3′ is complementary to the 5′ end of genomic RNA and sg RNA. Further, the 5′ ends of the gRNA and sgRNA promoter sequences of several *Sobemoviruses* begin with 5′ ACAA [1]. Similarly, the 3′ terminal sequence ends with GT 3′ or TGT 3′. It was proposed that the ACAA motif at the 3′ end of the negative strand might act as promoter or enhancer for replicase binding and initiation of progeny RNA synthesis [1], [30]. In order to study the importance of 5′ and 3′ end nucleotides in repair/replication, 1 nt, 3 nt and 5 nt were deleted at the position corresponding to 5′ end of the viral RNA in the SeMV icDNA clone. Similarly, 3 nt, 4 nt and 5 nt were deleted at the 3′ end as described in the methods section. These deletion mutants were agroinfiltrated onto *Cyamopsis* cotyledon leaves and samples were collected at 8 dpi and subjected to western blot analysis with CP antibodies. As shown in Fig. 7, significant amount of CP accumulation was observed when SeMV icDNA with 3 nt or 4 nt deleted from the 3′ end was infiltrated. However, only a small amount of CP was observed in a few plants when SeMV icDNA with 5 nt deletion from the 3′ end was infiltrated and none of these plants developed symptoms. On the other hand, deletion of 1 nt, 3 nt and 5 nt from the 5′ end did not result in complete loss of CP accumulation (Fig. 7).

Further, the virus was purified from the infiltrated leaves of all of these mutants (except 3′ UTR Δ 5 nt) to study the nature of nucleotide sequence at the 5′ and 3′ end of progeny viral RNAs. The RNA was extracted from purified virus and 5′ RACE (for 5′ deletion mutants) and 3′ RACE (for 3′ deletion mutants) was carried out as described in the methods section. Table 2 shows the 5′ end and 3′ end sequences obtained from independent clones. As apparent, the 5′ end sequence (5′ ACAA 3′) was efficiently restored when 1, 3, and 5 nt deletion mutants were infiltrated. Similarly the 3′ end was also efficiently repaired when 2 nt and 3 nt were deleted from the 3′ end of the SeMV icDNA. In all the sequences, the 3′ terminal TGT was restored. It was also observed that in some of the 3′ deletion mutants, the nucleotide at the 4^th^ position from the 3′ end was either changed to thymine or deleted. Similarly the 5^th^ nucleotide at the 5′ end was either restored to adenine or deleted. These results suggest that the nucleotide at the 4^th^ position from the 3′ end or the nucleotide at the 5^th^ position from the 5′ end is not crucial for infectivity. Interestingly no symptoms were observed when 3′UTR Δ 5′ nt deletion mutant of SeMV icDNA was infiltrated and poor CP accumulation was observed (Fig. 7) probably due to inefficient repair at the 3′ end. Attempts to isolate progeny virus from these plants were unsuccessful.

**Table 2:** 5′ and 3′ terminal sequence of progeny viral cDNAs of SeMV icDNA deletion mutants.

5′ Terminal deletion	5′ UTR terminal sequence*	3′ terminal deletion*	3′ UTR terminal sequence
WT	5′ ACAAAATAT3′	WT	5′CCAAATGT3′
	5′ ACAAAATAT3′		5′CCAAATGT3′
	5′ ACAAAATAT3′		5′CCAAATGT3′
1 nt Δ	5′ ACAAAATAT3′	2 nt Δ	5′CCAAATGT3′
	5′ ACAAAATAT3′		5′CCAAATGT3′
	5′ ACAAAATAT3′		5′CCAATTGT3′
3 nt Δ	5′ ACAAAATAT3′	3 nt Δ	5′CCAAATGT3′
	5′ ACAAAATAT3′		5′CCAATTGT3′
	5′ ACAA - ATAT3′		5′CCAA - TGT3′
			5′CCA - TTGT3′
5 nt Δ	5′ ACAAAATAT3′	4 nt Δ	5′CCAATTGT3′
	5′ ACAA - ATAT3′		5′CCAATTGT3′
	5′ ACAA - ATAT3′		5′CCAA - TGT3′
			5′CCAT - TGT3′

* The sequence obtained from 3 independent clones in each case is shown.

### Mutation of cleavage sites in polyprotein 2a and complementation analysis

The cleavage sites in SeMV polyprotein have been previously characterized with recombinant 2a and 2ab polyproteins from *E.coli* [11], [13]. In order to verify the role of these sites in SeMV infection and to study the importance of polyprotein processing in viral replication *in vivo*, site directed mutants of all four cleavage sites (E132A, E325A, E402A and E498A Fig. 1a) were generated in the *SeMV* icDNA clone. These cleavage site mutants were transformed into *Agrobacterium* and infiltrated into *Cyamopsis* plants separately. Symptom expression was monitored up to 30 dpi, on sets of 20 plants for each cleavage site mutant. None of the plants showed infection. Further western blot analysis carried out with cotyledon leaves 15 dpi on representative plants (3 each) did not show the presence of CP (data not shown). This result suggests that cleavages at all these sites are indeed crucial for viral replication/infectivity *in planta*.

**Figure 8:**
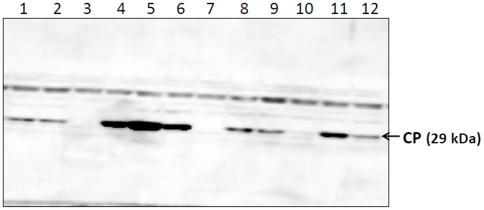
Western blot analysis of SeMV icDNA cleavage site mutants coinfiltrated with pEAQ ORF2. Each mutant was tested in 20 independent plants but three were used for western blotting. Lanes 1–3, SeMV icDNA E132A cleavage site mutant coinfiltrated with pEAQ ORF2; lanes 4–6, SeMV icDNA E325A mutant coinfiltrated with pEAQ ORF2; lanes 7–9, SeMV icDNA E402A mutant coinfiltrated with pEAQ ORF2; lanes 10–12, SeMV icDNA E498A mutant coinfiltrated with pEAQ ORF2.

In order to check whether these mutations could be complemented with wild type ORF 2 RNA, coinfiltration experiments were carried out. All the four cleavage site mutants were coinfiltrated with pEAQ ORF2 clone (contains only ORF 2 coding region and does not contain 5′ or 3′ non coding regions of SeMV). CP could be detected in the infiltrated leaves of all cleavage site mutants (Fig. 8). Absence of CP in lanes 3, 7 and 10 could be due to the fact that the infectivity of even the wild type SeMV icDNA is not 100%. Interestingly, the amount of CP accumulation was significantly high when coinfiltration was carried out with cleavage site mutant E325A and pEAQ ORF2 transformants (Fig. 8 lanes 4–6). These results suggest that ORF2 products could act in *trans* and promote the replication of cleavage site mutants when it is expressed from a high expression plasmid pEAQ. There was a non specific band above the CP band in all the lanes which was variable in different blots depending on the extent of washes given. Further it was observed that deletion of N-terminal hydrophobic domain (NΔ70) (membrane anchor) abolished the *trans* acting ability of ORF 2 products (data not shown) suggesting a crucial role of this domain in targeting the polyproteins to the site of replication.

## Discussion

It has been demonstrated that *in vitro* transcripts of full length cDNA clones are infectious in the case of *Rice yellow mottle virus*, *Cocksfoot mottle virus* and *Southern bean mosaic virus* [7], [10], [31]. However these transcripts showed varying degree of infection and were 5 fold less infectious than native viral RNA [10], [31]. In the present investigation, agroinfiltration approach was used to demonstrate that SeMV icDNA clone can infect *Sesbania* and *Cyamopsis* plants (Fig. 2 and 3). The efficiency of infection was optimized to be as high as 80% on *Cyamopsis* plants.

**Figure 9:**
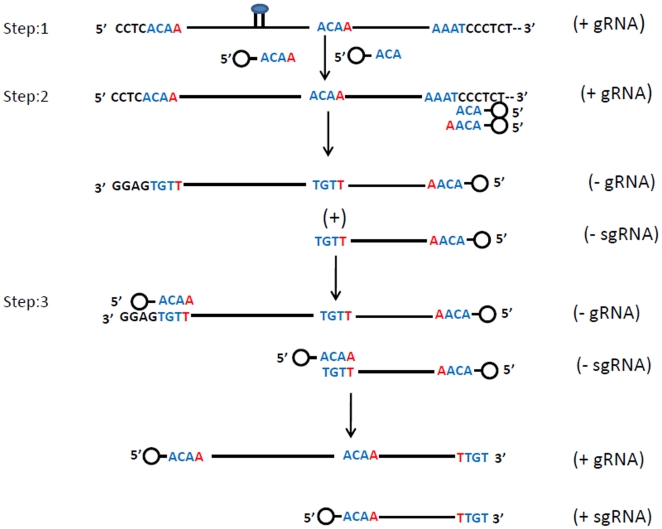
A possible mechanism for 5′ and 3′ end repair in SeMV. VPg is shown as a small circle at the 5′ end of the (+/−) gRNA, (+/−) sgRNA and primer nucleotides. The 5′ end of the (+) gRNA and (+) sgRNA begins with 5′ACAA3′ sequence. Step1, The VPg-ACAA or VPg-ACA primers could be synthesized by RdRp using unknown internal sequence element (shown as stem-loop) (presence of different VPg forms in the western blots supports this possibility). Step 2, these primers realign at the 3′ end of the (+) gRNA even in the absence of complementarity (Note that initial RNA formed from SeMV icDNA lacks complementarity with the VPg-ACA/VPg-ACAA primer at the 3′ end). Alignment or positioning of primers at the genomic termini could be determined by *cis* acting elements. The RNA chain could be elongated to synthesize full length negative strand or terminated prematurely to synthesize subgenomic length negative strand (the full length genomic negative strand in replicative form (ds gRNA) and (−) ss sgRNA or ds sgRNA are indeed detected in northern blots). Step 3, the VPg-ACAA/VPg-ACA primers align at the 3′ end of these negative stranded genomic and subgenomic RNAs which could be elongated to synthesize positive stranded genomic and subgenomic RNA respectively.

The nucleotide sequence analysis of the progeny RNAs from SeMV icDNA infected plants showed that the extra non viral 21 nt at the 3′ and 4 nt at the 5′ end were removed. Further the 5′ and 3′ ends were repaired to wild type or near wild type sequence (Fig. 6a & b). Analysis of the progeny viral RNA from the 5′ and 3′ end deletion mutants also confirmed that they were efficiently repaired. The tri or tetra nucleotide sequence at the 3′ end (5′ TGT 3′ or 5′ TTGT 3′) is complementary to that of the nucleotide sequence at the 5′ end of gRNA and sg RNAs (5′ ACA 3′/5′ ACAA 3′). These observations suggest that initially RdRp might bind to an internal sequence element yet to be identified and nucleotidylyate VPg resulting in the formation of 5′ VPg-ACAA 3′ or 5′ VPg-ACA 3′ primers (Fig. 9, Step1) . These primers could realign at the 3′ end of the genomic ( Step 2) or anti-genomic RNA (Step 3) to synthesize negative or positive strand viral RNAs (Fig. 9). Further, such a realignment is possible only if the deletion at 3′ end of SeMV icDNA is less than five nucleotides (Fig. 7). Further, it is interesting to note that there is no complementarity in the sequence of SeMV ic RNA transcript at the 3′ end and the primers VPg-ACA/VPg-ACAA (Fig. 9). However, positioning of the replicase complex at the initiation site is determined by several factors such as 5′ and 3′ *cis* acting elements [32], [33]. Our results suggest that such a *cis* acting element may be present in SeMV after four nucleotides from the 3′end. Analysis of 5′ terminal deletion mutants of SeMV icDNA beyond 5 nt might lead to the identification of *cis* acting elements also at the 5′ end of SeMV in future. Thus it is possible that during the repair process, replicase may overcome the requirement of complementarity in the nucleotide sequence of the template, at the initiation site. It was shown that viruses from *Picornaviridae* use protein primed initiation mechanism for repairing the genomic ends wherein the RdRp could uridylylate the VPg using the internal sequence of the template and could realign the nucleotidylated VPg at the genomic termini [33], [34], [35].

As shown in Fig. 5, different processing intermediates were identified in the SeMV icDNA infected leaf extracts suggesting that the polyprotein processing was indeed occurring at the expected cleavage sites [11]. In order to decipher importance of cleavage at these sites during virus replication/infectivity, all the four cleavage sites were independently mutated to alanine in the SeMV icDNA clone. As shown in the results section, mutation of any of the four cleavage sites (E132A, E325A, E402A and E498A) abolished CP accumulation/replication suggesting that cleavage at all the sites is indeed crucial for SeMV infection *in vivo*. Loss of infectivity upon mutation of E132 suggests that cleavage at this site by the protease domain is important for release of NΔ132Pro-VPg from membrane. This NΔ132Pro-VPg might perform proteolytic functions in *trans* as shown earlier [11], [15], [26] and these *trans* functions also are crucial for replication/CP accumulation. In *Potato leafroll virus* (genus *Polerovirus*), a similar cleavage site was identified and it was proposed that release of protease domain from the membrane may have a regulatory role [36]. The cleavage at E325 and E402 positions may be important for release of VPg for priming the replication. Western blot analysis showed the presence of the fully processed VPg apart from Pro-VPg and VPg-p10 suggesting that cleavages at both ends of the VPg had occurred (Fig. 5). Mutation of E498 site also abolished the viral replication suggesting that release of p8 from the rest of the polyprotein 2a may be important. The p8 was shown to be an RNA binding protein and may be required for specific binding to genomic RNA and targeting it to the site of replication. The detection of Pro-VPg-p10 band in western blot analysis suggests that the cleavage between p10-p8 may be important for the release of p8 along with viral genome at the replication site. The loss of infectivity may also be due to changes in the conformation of the polyprotein upon mutation of the residue at the cleavage site. However, earlier studies [11] on cleavage site mutants of the poly proteins have shown that while the cleavage at the site of mutation does not occur, cleavages at other sites remain unaffected suggesting that changes in the amino acid sequence itself may not be responsible for lethality. However, a more detailed analysis on the lethality due to mutation of the cleavage site amino acid will be tested in future by introducing conservative mutations.

Coinfiltration analysis of cleavage site mutants with SeMV ORF 2a/2ab showed that cleaved 2a/2ab products could act in *trans* and restore the replication of these mutants (Fig. 9). Interestingly ORF 2a/2ab products complemented the cleavage site mutant E325A more efficiently than any other cleavage site mutants (Fig. 9). Earlier studies have shown that mutation of cleavage site E325 in the ΔN70 polyprotein 2a results in accumulation of ΔN70Protease-VPg, which is an active form of the protease [11], [15]. Further *in vitro* studies also showed ΔN70Protease-VPg could act in *trans* and could cleave the polyprotein 2a at E325 and E402 positions [11], [15], [26]. A significant accumulation of full length Pro-VPg was also observed in membrane fractions of *SeMV* icDNA infiltrated leaf samples (Fig. 5). It is therefore possible that accumulation of enzyme (Protease-VPg expressed from icDNA E325A and pEAQ ORF2) at the site of viral replication may result in efficient processing of polyproteins and release of VPg and other domains in sufficient quantities for virus replication. Furthermore, no complementation was observed when coinfiltration was carried out with pEAQ-NΔ70 ORF 2 suggesting that N-terminal membrane anchor domain is crucial for this complementation. These results emphasize the necessity of the polyproteins to first target themselves to membrane (where replication occurs) via the membrane anchor domain prior to their processing into functional domains. Such a targeting of the polyproteins to the membranes would ensure the release of processed products at the site of replication.

In summary, this paper describes the construction and optimization of conditions for agroinfiltration of SeMV infectious clone on *Cyamopsis tetragonoloba* plants. Based on mutational analysis of 5′ and 3′ ends of SeMV genome and detection VPg in different forms, a possible genomic end repair mechanism was proposed. Analysis of cleavage site mutants showed that cleavage at all the four sites previously characterized with recombinant proteins in the polyprotein 2a/2ab are essential for infection. Further, products of processing are functional only when released at the site of replication.

## Materials and Methods

### Construction of SeMV icDNA Clone

SeMV full length cDNA clone was initially constructed in pBluescript SK+ vector and later subcloned into pRD400 binary vector (Fig.S1). The schematic representation of the infectious cDNA (icDNA) clone construction is shown in Fig.S1. The *Nos* terminator (T) was PCR amplified from PVA icDNA [37] clone using appropriate sense and anti sense primers (Table 1) and cloned at *Sma*I site of pBluescript SK+ (Fig.S1). SeMV full length cDNA was PCR amplified from pFX37 SeMV full length clone (Lokesh G.L., unpublished clone) using sense and antisense primers corresponding to 5′ and 3′ ends of the genome (Table 1) and subcloned at *Eco*RV site of pBluescript SK+ (T) (Fig.S1.) The SeMV full length genome sequence is available at GenBank with Accession number AY004291. Double 35S promoter was PCR amplified from PVA icDNA [37] using appropriate sense and antisense primers (Table 1 and cloned at *Stu*I site located at the 5′ end of SeMV cDNA in pBluescript SK+ SeMV-(T) clone Fig.S1. The sequence for 35S promoter is available at GenBank with Accession number FN398079.1. The Ribozyme sTobRV (satellite RNA of *Tobacco ring spot virus*)(Fig.S1) was PCR amplified from pCass4RZ binary vector [19] using primers (Table 1) and subcloned at *Sma*I site located at the 3′ end of the SeMV in pBluescript SK+ 35S-SeMV-(T) clone (Fig.S1) (The sequence of the Ribozymes is 5′ *TCTAGA*GATACCCTGTCACCGGATGTGTTTTCCGGTCTGATGAGTCCGTGAGGACGAAACAGGACTGTCCTGCAG*AGGCCT*3′) [19]. In each cloning step orientation was confirmed by PCR using appropriate primers (Fig.S1). The final clone was confirmed by sequencing with T3 FW, T7 FW and 35S FW primers. The icDNA cassette from pBluescript SK+ vector was released by digestion with *Bam*HI and subcloned at *Bam*HI site of pRD400 vector [38]. The clone was confirmed by restriction digestion with *Kpn*I. This clone was named as SeMV icDNA.

All the DNA sequences used in the construction of SeMV icDNA are published and/or available at GenBank with accession numbers given above.

### Site directed mutagenesis

The cleavage site mutants, 5′ and 3′ end deletion mutants were generated by PCR based site directed mutagenesis method as described by Weiner *et al* [39]. PCR was performed using pBluescript SK+ SeMV icDNA template with appropriate sense and antisense primers (Table 1) and Phusion polymerase (Finzymes). The PCR product was digested with *DpnI* to remove templates followed by transformation into DH5α competent cells. Plasmids were isolated from colonies and screened by digestion with appropriate restriction enzymes (Table 1). The mutations were confirmed by DNA sequencing. The inserts were released with *Bam*HI digestion and subcloned at the same site of pRD400 binary vector.

### Cloning of pEAQ-ORF2 and pEAQ-NΔ70 ORF2

The SeMV ORF 2 and NΔ70 ORF 2 were PCR amplified from the SeMV cDNA template using appropriate sense and antisense primers (Table 1) and Phusion polymerase. The PCR products were cloned at *SmaI* site of pEAQ-HT vector [40]. The clones were confirmed by sequencing.

### Agroinfiltration protocol

Agroinfiltration was carried out essentially as described by Eskelin *et.al.*, [37]. Briefly, *Agrobacterium tumefaciens* strain C58C1 [41] containing the helper plasmid pGV2260 was transformed with the binary vector constructs. Transformation was carried out by electroporation (voltage 1.44 kV, conductivity 25 µF, and resistance 100–200 Ω). After electroporation, cells were grown in plain LB medium for 3–4 hours at 28°C with vigorous shaking. The cells were harvested by centrifugation at 3000 g for 5 min and platted on LB agar plates containing kanamycin, carbenicilin and rifampicin (100 µg/ml each) and incubated at 30°C for 48 hours. Single colony was inoculated to 3–50 ml of LB medium containing 10 mM MES pH 6.3 (2-(N-morpholino) ethanesulfonic acid) and 20 µM of acetosyringone (3′-5′-dimethoxy-4-hydroxyacetophenone) and antibiotics 100 µg/ml and grown at 30°C with shaking (200 rpm) until optical density at 600 nm (OD_600_) reached 0.6–0.8. The cells at this stage were harvested by centrifugation at 3000 g for 5 min and the pellet was washed with milli-Q water, followed by resuspension in induction buffer (10 mM MES pH 6.3, 10 mM MgCl_2_, and 150 µM acetosyringone). The suspension was diluted with induction buffer to desired density (OD_600_ 0.05 to 1.2) and incubated at room temperature for 3–4 hours. The cotyledon leaves of *Sesbania grandiflora* or *Cyamopsis tetragonoloba* plants were chosen for infiltration. Leaves to be infiltrated were turned upside down and a small prick was made with a needle in the middle of intended infiltration area and the bacterial suspension was injected at this position with 1 ml syringe without needle.

#### Coinfiltration

Coinfiltration experiments were carried out with *Agrobacterium* from each transformant at OD_600_ 0.4 (*SeMV* icDNA cleavage site mutant+pEAQ ORF2) in such a way that the combined final OD_600_ was 0.8. This mixture was infiltrated onto plants as described above.

### Western blotting

100 mg of leaf sample was homogenised in 500 µl buffer 50 mM phosphate buffered saline (PBS) pH 7.4. 20 µl of the homogenised sample (containing 400 µg of protein according to absorbance at 280 nm) was used for SDS-PAGE followed by western blot analysis. The SDS-PAGE was carried out at 125 V, for 2 hours. After SDS-PAGE proteins were electro-blotted on to PVDF membranes by applying a current of 100–150 mA for 2–3 hours. Membrane was blocked with 5% skimmed milk solution (in PBS) for one hour followed by incubation with primary antibody for one hour (rabbit polyclonal SeMV CP or VPg antibodies were used in 1∶5000 ratio). Blot was washed with phosphate/Tris Buffered saline pH 7.5, 0.1% tween 20 (PBST/TBST). Finally the blot was incubated with secondary antibody (goat polyclonal anti rabbit IgG HRP conjugate antibodies were used in 1∶10,000 ratio) for one hour followed by washing with PBST/TBST for one hour (three times 20 min each). The blot was developed using ECL reagent (Millipore).

### Northern analysis

Northern blotting was carried out as described previously [16]. Briefly, total RNA was extracted from 100 mg of cotyledon leaves using Trizol method. The RNA (2–4 µg) was run on 0.8% TBE agarose gel and transferred to Nylon membrane by electro blotting (150–200 mA for 2 hours). The blot was exposed to UV for cross linking and blocked with hybridization buffer containing 2×SSC, 50% formamide, 1.3×Denhardt's reagent (50×Denhardt's reagent contains 1% bovine serum albumin (BSA), 1% polyvinyl pyrrolidone (PVP), 1% Ficoll), 100 µg/ml salmon sperm DNA, 7% SDS and 0.1% sodium-N-lauroyl sarcosine detergent at 65°C for 3 h. The probe (1.5×10^6^ cpm/ml) was added to the hybridization buffer not containing salmon sperm DNA and the probe was allowed to hybridize with the immobilized RNA at 68°C for 14 h. The blot was washed with 2×SSC, 1×SSC and 0.2×SSC containing 0.1% SDS at 65°C. The blot was finally exposed to a phosphor-imager and analyzed by Fuji-film LAS 9000 instrument.

### RT-PCR

The total RNA was extracted by trizol method and about 2 µg of RNA was annealed to DNA oligo nucleotide (40 pmol) by heating at 72°C for 5–10 min and immediately chilling on ice. This was followed by addition of 1× MuLV buffer, 1 mM rNTPs, 1 U/µl RNase inhibitor, 1 µl of MuLV RT (200 U/µl) to the total reaction mixture (20 µl). The reaction mixture was incubated at 37°C for 5 min followed by incubation at 42°C for one hour. The PCR was carried out using 1 µl of RT-reaction mixture in 50 µl PCR cocktail containing appropriate sense and antisense primers, dNTPs and Phusion polymerase. The CP sense and RdRp anti-sense primers were used for detection of viral RNA in SeMV icDNA infiltrated and systemic leaves.

### Poly A tailing of genomic RNA (For 3′ RACE)

Poly A tailing of genomic RNA was carried out with 10 µg of gRNA using poly A polymerase (Ambion) as described by the manufacturer. After the reaction, the RNA was extracted with Trizol, Chloroform and precipitated with isopropanol. The RNA pellet was dissolved in nuclease free water and used for RT-PCR with appropriate primers.

### Addition of poly dA at the 3′ end of cDNA (For 5′ RACE)

After reverse transcription (using P1 reverse primer) the reaction mixture was treated with RNase A (1 µg) for 15 min at 37°C and 15 min at 45–50°C to remove the RNA template. The cDNA was extracted with phenol and chloroform and precipitated with isopropanol. The cDNA was then incubated with terminal transferase in the presence of dATP (130 pmol). The second strand synthesis and amplification was carried by PCR with oligo dT sense and P1 gene specific antisense primers (Table 1). The high fidelity Phusion polymerase (Finzymes) was used in the PCR reaction.

### Preparation of crude membrane fraction

Cotyledon leaves infected with SeMV icDNA were collected 8–10 days post infiltration (dpi) and used to isolate crude membrane fraction. Five g of cotyledon leaves were ground in 20 ml of buffer containing 20 mM Tris-HCl pH 7.5 and10 mM MgCl_2_. The leaf extract was passed through muslin cloth and the flow through was centrifuged at 2000 g for 10 min to remove debris. The supernatant was centrifuged at 10,000 g for 10 min and the pellet was resuspended in 2–3 ml of buffer. The supernatant obtained after centrifugation at 10,000 g was again centrifuged at 25,000 g for 30 min and the pellet was resuspended in 2–3 ml of buffer. These pellet fractions were used for western blot analysis.

## Supporting Information

Figure S1 **A schematic representation of the SeMV icDNA construction.** Initially the Nos terminator (red box) was cloned at SmaI restriction site and the orientation was confirmed by PCR using Nos forward and T3 reverse primers. The SeMV cDNA (purple box) was cloned at EcoRV site and the orientation was confirmed by PCR with CP forward and Nos reverse primers. The double 35S promoter (green arrows) was cloned at the StuI site at the 5′ end of the SeMV cDNA and the orientation was confirmed by PCR with T7 forward and 35S reverse primers. The ribozyme (yellow box) was cloned at the SmaI site at the 3′ end of the SeMV cDNA and orientation was confirmed by PCR with ribozyme forward and Nos reverse primers. The entire cassette 2×35S-SeMV cDNA-Rz-Nos was released by digestion with BamHI and subcloned into pRD400 vector.
(TIF)

Figure S2 **Comparison of agroinfiltration efficiency in** ***Sesbania*** **and** ***Cyamopsis*** **plants.** *Agrobacterium* containing pEAQ-GFP at an OD_600_ of 0.6 was infiltrated onto (a) *Sesbania* plants (b) *Cyamopsis* plants.
(TIF)
