# Supplementary figures and images for: Sesbania Mosaic Virus (SeMV) Infectious Clone: Possible Mechanism of 3′ and 5′ End Repair and Role of Polyprotein Processing in Viral Replication
Source: PLoS One. 2012 Feb 15;7(2):e31190. doi: 10.1371/journal.pone.0031190 (PMC3280281; doi:10.1371/journal.pone.0031190)

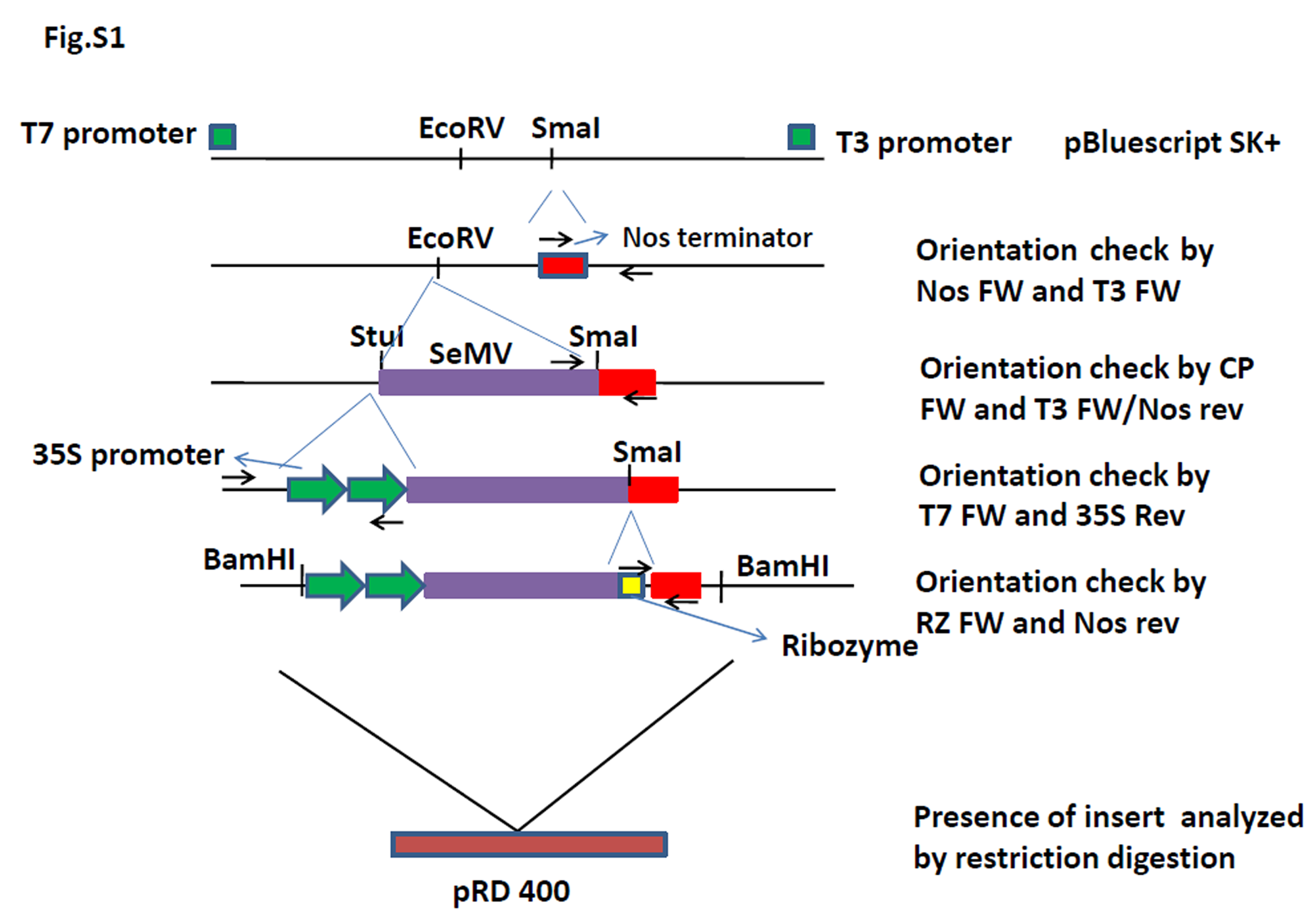

Supplement: Figure S1 — A schematic representation of the SeMV icDNA construction. Initially the Nos terminator (red box) was cloned at SmaI restriction site and the orientation was confirmed by PCR using Nos forward and T3 reverse primers. The SeMV cDNA (purple box) was cloned at EcoRV site and the orientation was confirmed by PCR with CP forward and Nos reverse primers. The double 35S promoter (green arrows) was cloned at the StuI site at the 5′ end of the SeMV cDNA and the orientation was confirmed by PCR with T7 forward and 35S reverse primers. The ribozyme (yellow box) was cloned at the SmaI site at the 3′ end of the SeMV cDNA and orientation was confirmed by PCR with ribozyme forward and Nos reverse primers. The entire cassette 2×35S-SeMV cDNA-Rz-Nos was released by digestion with BamHI and subcloned into pRD400 vector. (TIF) [file pone.0031190.s001.tif]

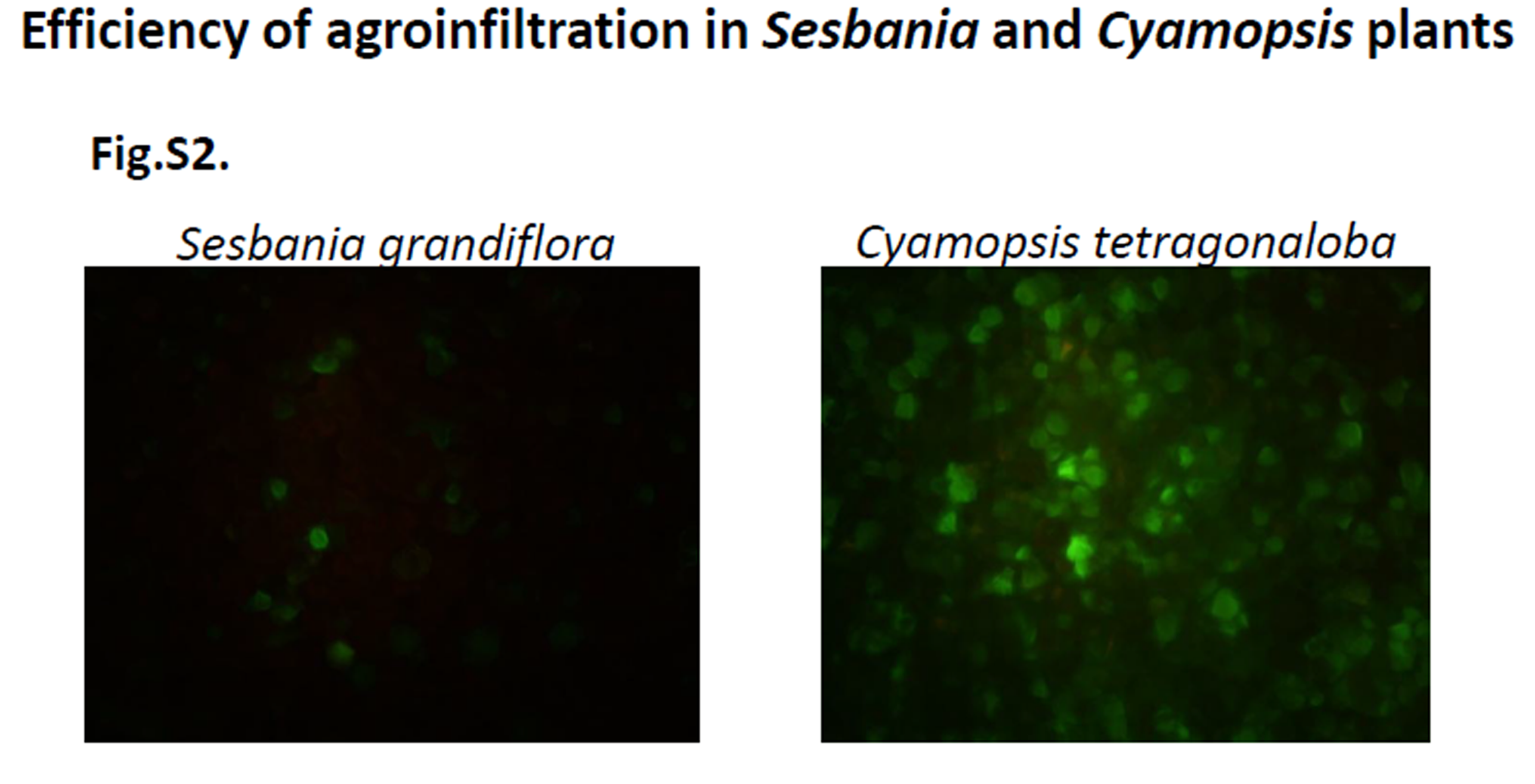

Supplement: Figure S2 — Comparison of agroinfiltration efficiency in Sesbania and Cyamopsis plants. Agrobacterium containing pEAQ-GFP at an OD600 of 0.6 was infiltrated onto (a) Sesbania plants (b) Cyamopsis plants. (TIF) [file pone.0031190.s002.tif]
